# It’s Time to be disgusting about COVID-19: Effect of disgust priming on COVID-19 public health compliance among liberals and conservatives

**DOI:** 10.1371/journal.pone.0267735

**Published:** 2022-05-12

**Authors:** Kellen Mermin-Bunnell, Woo-kyoung Ahn

**Affiliations:** Department of Psychology, Yale University, New Haven, CT, United States of America; University of Connecticut, UNITED STATES

## Abstract

The COVID-19 pandemic is far from over, in part due to poor adoption of public health mitigation measures in the U.S. and the continued spread of the Delta and Omicron variants. Current public health messaging used in the U.S. could be improved to better combat mistrust about COVID-19 and its mitigation measures, especially vaccines. We propose that a disgust-inducing public health campaign will be more effective than current approaches, primarily among conservatives, who are both sensitive to moralized disgust and are less compliant with U.S. public health guidelines. Using a convenience sample across two studies (n = 1610), we found that presenting disgusting images related to the COVID-19 pandemic increased public health compliance more among conservatives than among liberals. Among unvaccinated conservative participants, disgusting images significantly increased willingness to be vaccinated compared to less disgusting images of COVID-19 or perks offered for COVID-19 vaccines. Using disgusting images for public health messaging has the potential to improve compliance among conservatives and accelerate the end of the COVID-19 pandemic in the U.S.

## Introduction

The COVID-19 pandemic has devastated the globe. Controlling community transmission is critical for stopping the development of more virulent and infectious variants. As healthcare providers struggle to treat the overwhelming influx of COVID-19 patients, public health experts urge communities to adhere to social distancing and mask-wearing guidelines as well as to receive COVID-19 vaccines. A public health information campaign that employs rigorously tested psychological strategies could reduce the spread of SARS-CoV-2 and associated mortality.

Misinformation, however, has fueled resistance to public health guidelines, particularly among some sub-populations, and has characterized the U.S. response to the pandemic. In January of 2021, only 52% of Americans reported avoiding contact with other people, 59% avoiding crowded places, and 80% wearing a face mask when outside the home [1]. Additionally, vaccine hesitancy is prevalent: 29% of Americans said in January 2021 that they were somewhat or extremely unlikely to get the COVID-19 vaccine when made available to them [2], and as of March of 2022, the percentage of the U.S. population who have been fully vaccinated is only 65.9% despite it is readily available across the country. Disseminating COVID-19 public health information campaigns to as many people as possible is important, but strategically targeted campaigns that are evidence-based both from a medical and a psychological perspective could appeal to specific populations and improve adherence to public health mitigation measures.

In December of 2020, *The New York Times* published an Opinion piece titled, “It’s Time to Scare People About COVID-19” [3]. The author, Dr. Elisabeth Rosenthal, draws an analogy to an anti-smoking campaign Tips from Former Smokers (Tips campaign) that used terrifyingly graphic videos or pictures, like a woman talking through a hole in her throat, gasping for air, and testimonials from former smokers who showed the physical effects they had experienced (e.g., mottled scars from cardiovascular surgeries and removal of half the lower jaw due to oral cancer). Such fear-inducing campaigns turned out to be successful: the antismoking campaign increased quit attempts by 12%, to over 100,000 [4]. Rosenthal argued we should do the same with COVID-19. For instance, hard-hitting, graphic images of COVID-19 patients could prompt behavior change by appealing to strong emotions. The present study aims to investigate the efficacy of a disgust intervention on compliance with the COVID-19 safety guidelines published by the U.S. Centers for Disease Control and Prevention (CDC) [5].

There are several reasons why a disgust intervention could be effective. Disgust is a powerful, purity-based emotion, deeply rooted in evolutionary adaptations [6]. Pathogen avoidance and exclusion of potentially harmful infected or impure group members are universal and grounded in biology [7]. In fact, several studies have demonstrated that individual differences in sensitivity to disgust predict the extent to which one engages in healthy behaviors or fears infectious diseases. For instance, participants who found scenarios such as “you see maggots on a piece of meat in an outdoor garbage pail” to be highly disgusting were more concerned about the threat of the Ebola [8] and Zika viruses [9]. In relation to the current pandemic, those who were more sensitive to disgust tended to be more fearful of contracting COVID-19 [10] and were more willing to engage in preventive health behaviors such as handwashing and wearing face masks [11]. Even pre-pandemic disgust proneness measured four years prior to the COVID-19 pandemic predicted increased anxiety about contracting SARS-CoV-2 as well as safety behaviors [12].

Additionally, disgust induces moral dumbfounding: despite the lack of reasoning people are able to provide for their moral disgust judgments, it drives moral behavior. [13, 14]. Indeed, pathogen-based disgust has been shown to predict utilitarian moral judgments [15]. Public health guidelines are, generally, morally utilitarian—aimed at an outcome-based maximal reduction of morbidity and mortality; therefore, inducing pathogen-based disgust could directly impact moral judgment and behavior in the context of a pandemic.

In particular, a disgust intervention could be effective because conservatives tend to value the moral foundation of purity with much higher regard than liberals do [16–18], and Republicans comply less with COVID-19 guidelines than Democrats do [19, 20]. For instance, the U.S. counties that voted for Donald Trump over Hillary Clinton in the 2016 presidential election exhibited significantly less social distancing in 2020 than the counties that voted for Hilary Clinton [21]. Additionally, social conservatism is associated with pathogen disgust sensitivity [22]. Conservatives are also higher in religiosity, indicating that they would be likely to engage in impartial beneficence when primed to make utilitarian moral judgments [23, 24]. Consequently, a disgust intervention appears to be a particularly suitable method to boost compliance because conservatives tend to care more about protecting moral purity.

However, disgust may also decrease preventive health behaviors because it can make people avoid engaging with additional disgust-inducing stimuli. For instance, participants who felt disgust when contemplating the spread of an infectious disease were less likely to engage in information-seeking about the outbreak [25]. Yet the knowledge-avoiding component of disgust is likely to be less relevant in the context of a lived pandemic in which the news, government, and social media are publishing copious amounts of information about how to prevent infecting oneself and others. More challengingly, however, several recent studies found that higher levels of disgust sensitivity are associated with more negative attitudes about vaccines [26–28]. For instance, those who were more sensitive to disgust were more skeptical of the importance and safety of vaccinations.

This double-edged nature of disgust has been well-articulated and empirically demonstrated in a mediation analysis examining the association between disgust sensitivity and flu vaccine uptake [29]: “Since vaccines prevent infection, those with heightened disgust sensitivity might hold more positive attitudes towards vaccines so as to prevent themselves from infection. In contrast, those with heightened disgust sensitivity could hold more negative attitudes towards vaccines perhaps because they view the vaccines themselves as contaminants.” Luz, Brown, and Struchiner found that these apparently opposing associations may coexist because, while those with heightened disgust sensitivity were more likely to have received flu vaccines, increased disgust sensitivity could also decrease vaccine uptake by increasing negative attitudes towards vaccines (e.g., increased misconceptions that vaccines cause children to develop Autism Spectrum Disorder).

To summarize, existing literature suggests mixed results in relation to the role of disgust in promoting healthy behaviors. Previous studies, however, focused on the role of people’s baseline sensitivity to disgust in public health behaviors rather than how presenting disgusting images as in the Tips campaign would induce preventive health behaviors. On one hand, the findings showing that heightened disgust sensitivity is associated with increased vaccine aversion could, at face value, imply that those who are highly disgust-sensitive may become more resistant to being vaccinated after seeing disgusting images. However, we propose that the disgusting images which specifically convey the disgusting aspects of infection with SARS-CoV-2 (e.g., dead bodies of COVID-19 victims) are more disgusting than the mRNA and water that make up vaccines or the process of receiving a vaccine via injection with a small needle. When the images presented highlight the disgusting nature of COVID-19, a disgust intervention may overpower vaccine resistance even among those who are highly sensitive to disgust—the disgust prompted by COVID-19 could be stronger than disgust prompted by vaccines, thus improving vaccine uptake to reduce risk of infection and severe disease.

Several studies examined the effects of presenting disgusting images on behavioral hygiene or willingness to be vaccinated and found results consistent with our predictions. In a field experiment, when a disgust-based poster depicting the chain of disease transmission using a picture of “a long bread roll containing feces as a filling” was posted in a washroom, people were more likely to wash their hands than when a poster communicating the same message without the disgust-eliciting stimulus was used [30]. Trujilo and colleagues found direct support for the disassociation between effects of disgust sensitivity and effects of disgust interventions [27]. On one hand, the higher moral purity [16] the participants in the study showed, the more they viewed MMR vaccines as contaminants and endorsed misinformation about MMR vaccines. On the other hand, when those with high levels of moral purity read disgusting descriptions of measles infections (e.g., “sticky bluish-white bumps had infiltrated the one-year-old’s cheeks”), the tendency to endorse vaccine misinformation was diminished [27].

Thus, we predict that presenting disgusting images of COVID-19 will push those who are more sensitive to disgust and purity violations to endorse behaviors that will counteract more disgusting consequences (i.e., the actual disease) than vaccinations by prompting them acknowledging the threat of the pandemic. To test this prediction, we examined the impact of a disgust intervention on CDC COVID-19 guideline adherence. In Study 1, participants viewed either disgusting or non-disgusting pictures and news headlines about COVID-19 and answered 5 sets of questions measuring compliance with the CDC’s COVID-19 guidelines (e.g., mask wearing, receiving a vaccine). We hypothesized that disgusting pictures would have a greater impact on the public health compliance of conservative participants than liberal participants either because the liberals would already be highly compliant and/or because disgust-induced purity moralization would be valued more by the conservatives.

In Study 2, we tested the effectiveness of an intervention currently being used to encourage people to receive COVID-19 vaccines. Many businesses are offering incentives to those who choose to get vaccinated. For example, when the vaccines were first introduced, some bars gave out “a shot for a shot”—a free shot of liquor to anyone who presented a completed vaccine card; Krispy Kreme, a donut conglomerate, offered fully vaccinated Americans a free donut every day for the entirety of 2021 [31]. Between December 21^st^ and December 31^st^, 2021, New York City offered $100 to people who received their booster shot.

But the reasons people have for feeling hesitant about receiving COVID-19 vaccines tend to be fundamental concerns about accelerated development in laboratories and rapid clinical trial processes, general mistrust of the government, or ideological beliefs about the COVID-19 pandemic or vaccines in general. Therefore, vaccine hesitancy is unlikely to be due to a lack of immediate, tangible rewards to those who receive vaccines. Indeed, people could see the perks offered to those getting a vaccine as indicative of the vaccines being dangerous; if they were safe, people would receive them without needing any additional perks [32]. Thus, we investigated whether these incentives increase or decrease willingness to receive a COVID-19 vaccine. We predict that our disgust intervention will increase intent to get vaccinated more than perks will by triggering basic instincts as opposed to transient perks.

### Pretest

A pretest was conducted to select especially disgusting and especially non-disgusting images for use in both Studies 1 and 2. In the pretest, 30 participants, who were later excluded from participating in the main experiments, were recruited from Amazon’s Mechanical Turk. Of these, 15 were liberal and 15 were conservative.

A set of 22 pictures and headlines were initially selected from newspaper webpages and the Google Images search engine. These images were selected to span various levels of disgust, ranging from highly disgusting to not at all disgusting. Because the vast majority of photographs used in the news did not include disgusting imagery, we searched for disgusting images using specific keywords (e.g. “COVID-19 toes” or “COVID-19 lungs”).

Photographs were paired with headlines, and participants were asked to rate each on how disgusting the image was using a sliding scale that ranged from “not at all disgusting” (0) to “extremely disgusting” (10). Participants then completed demographic questions and an image recognition attention check. All participants passed the attention check. There was no significant difference between the liberal participants (M = 2.38) and the conservative participants (M = 2.21) on the disgusting ratings, p = .78.

The average disgust rating was calculated for each of the 22 photographs in the pretest. Using these ratings, we selected the 5 most disgusting and the 5 least disgusting photos—these together were the 10 images used in Studies 1 and 2. The disgusting images included photos of COVID-19 toes (https://www.nytimes.com/2020/05/01/health/coronavirus-covid-toe.html), lungs of patients who had been infected with SARS-CoV-2 (https://www.thelancet.com/journals/lanres/article/PIIS2213-2600(20)30361-1/fulltext), and gravediggers burying bodies of COVID-19 victims (https://www.scientificamerican.com/article/the-bodies-of-people-who-died-from-covid-19-may-still-be-contagious/). The non-disgusting images included doctors and patients wearing masks (https://www.mibluesperspectives.com/2020/06/24/how-doctors-offices-are-keeping-patients-safe-during-covid-19/), physicians wearing PPE (https://www.dw.com/en/coronavirus-in-germany-medical-students-step-up-to-fight-covid-19/a-5301994), and patients preparing to receive a COVID-19 vaccine (https://www.healthline.com/health-news/why-some-black-and-latinx-people-are-reluctant-to-get-the-covid-19-vaccine). The stimuli for both the pretest and the main experiments are available from the authors upon request.

## Study 1

Study 1 examined the effect of disgusting and non-disgusting images on willingness to comply with CDC’s COVID-19 guidelines among liberals and conservatives.

### Methods

#### Participants

Participants were recruited between February 26^th^, 2021 and March 1^st^, 2021 when vaccines for COVID-19 were available mostly for priority populations such as health workers. Based on similar studies (e.g., [18], with 74 Republicans), the target sample size was 90 conservative and 90 liberal participants in each condition. In case participants’ pre-declared political orientation changed, we recruited 100 in each condition for each political orientation.

Using convenience sampling, 400 participants all located in the U.S. were recruited from Amazon’s Mechanical Turk. Of these, 200 slots were available for those who previously declared their US political affiliation to be conservative, and 200 were available for those who previously declared it to be liberal. Of the 399 participants who completed the experiment, 6 failed to pass the attention check (see below) and 15 turned out to be neither conservative nor liberal when responding to our demographic questions (see below). These 21 participants were not included in data analyses. The data from the remaining 378 participants were used in the data analyses.

Because it is unclear how and when Amazon’s Mechanical Turk assessed the political affiliation, and the participants’ political affiliations may have changed since the last time they indicated them for Mechanical Turk, participants were asked at the end of the study to indicate their political ideology on a 7-point scale ranging from “strong conservative” to “strong liberal” similar to [18]. Those who selected strong to moderate liberal were classified as “Liberal” and those who selected strong to moderate conservative were classified as “conservative”. Also, given the recent findings that political conservatism (i.e., voting for Trump vs. Clinton) determines social distancing [24], participants were also asked to indicate their political party (e.g., Strong / Weak / Independent Democrat, Strong / Weak / Independent Republican, Libertarian, non-partisan). Those who selected strong, weak, or independent Democrat were classified as “liberal”, those who selected strong, weak, or independent Republican were classified as “conservative”, and all others were classified as “other”. The responses to these two questions were combined as follows: Those who were classified as “conservative” in either question and not as “liberal” (e.g., other or middle of the road) were categorized as “conservative” (n = 165). Those who were classified as “liberal” in either question and not as “conservative” were categorized as “liberal” (n = 213). The rest of the participants (n = 15; i.e., those who selected independent, or middle of the road on both questions, those who were classified as liberal in one question and conservative in the other question) were not included in the data analyses. (See the S1 File for the analyses including those who failed the attention check and the analyses treating the political party affiliation and political ideology as continuous variables. In short, the general pattern of the results reported below did not change.)

Of the 378 participants whose data were used for the analyses, 50.3% were female, 35.2% had received a high school degree or some college education, and 45.5% received a bachelor’s degree. The participants were 85.2% White, 6.9% Asian, and 4.5% Black. None of these demographic variables significantly interacted with any of the independent variables, and thus they are not included in the analyses. The data have been deposited at https://osf.io/t7d6a/.

The study was approved by Yale University IRB. Participants provided written consent.

#### Design and procedure

The 10 pictures and headlines about COVID-19 selected through the pretest were used as stimuli. Participants were randomly assigned to either the disgust condition (n = 187), in which five pairs of disgusting pictures and headlines were presented, or the nondisgust condition (n = 191), in which five pairs of non-disgusting pictures and headlines were presented. On each trial, the headline was presented on the top, followed by the corresponding picture. In order to encourage participants to remain engaged while reading the headlines and processing the pictures, participants were asked to rate how relevant the picture was to the headline on each trial. Trial order was randomized across participants.

After viewing all five trials, participants answered questions about their likelihood to engage in public health-recommended pandemic behaviors. The recommended behaviors were taken from COVID-19 pandemic safety guidelines on the CDC’s website in the U.S. The first set of questions measured participants’ likelihood to wear a mask in each of six indoor public spaces (grocery store, office/at work, pharmacy, non-essential goods retail establishment, restaurant, coffee shop). The second set measured participants’ likelihood to wear a mask in each of five outdoor public spaces (outdoor party/gathering, walking down a busy sidewalk, waiting in line/in front of a public area, outdoor restaurant/coffee shop, in a park/dog park). The third set measured the likelihood that they would wash or sanitize their hands in each of eight situations (before touching your face, before eating or preparing food, after using the restroom, after leaving a public place, after blowing your nose, coughing, or sneezing, after caring for someone sick, after cleaning a frequently touched surface). The fourth and the fifth set each had only one question, asking how important frequent COVID-19 testing is for public health, and how likely they are to get a COVID-19 vaccine when one is available to them. In all of these questions, participants responded by adjusting a sliding scale that ranged from 0 (Definitely not) to 100 (Definitely).

Afterwards, all participants were presented with 10 pictures, 5 of which they had previously seen and 5 from the other condition and were asked to check images that they had seen earlier. Those who failed to recognize at least 3 of the 5 pictures were the six participants who were excluded from the data analyses for failing the attention-check.

### Results

The sub-questions within each of the indoor mask-wearing, outdoor mask-wearing, and the handwashing set all had high reliabilities (all Cronbach’s alpha’s > .89). Thus, for each participant, the average score within each of these sets was computed. These three average scores and the fourth and the fifth dependent measures (importance of frequent COVID-19 testing and vaccination) also showed a high reliability (Cronbach’s alpha = .82). These five scores (see Table 1) were averaged for each participant and are henceforth called compliance scores. The main analyses are based on these compliance scores because in order to effectively mitigate the spread of SARS-CoV-2, people need to follow all of these public health measures. Following only one of these guidelines would not be sufficient to significantly reduce transmission of the virus. Thus, the composite measures would be a better index for the public health compliance rather than each individual measure.

**Table 1:** Descriptive statistics for the five sets of dependent measures for conservative and liberal participants in each condition.

	Conservative				Liberal			
Dependent Measures	Disgust Condition		Non-disgust Condition		Disgust Condition		Non-disgust Condition	
	*M*	*SD*	*M*	*SD*	*M*	*SD*	*M*	*SD*
Wearing masks indoors	84.26	19.88	69.37	33.29	93.02	15.08	95.44	8.72
Wearing masks outdoors	54.05	30.47	39.15	34.82	78.19	25.99	79.12	23.99
Cleaning hands	85.37	16.59	78.71	21.78	83.43	18.77	88.54	16.70
Testing for COVID-19	75.06	28.09	63.43	32.66	88.93	17.91	91.27	15.43
Receiving a COVID-19 vaccine	63.11	39.71	52.76	39.07	83.08	28.16	86.78	24.79

**Fig 1:**
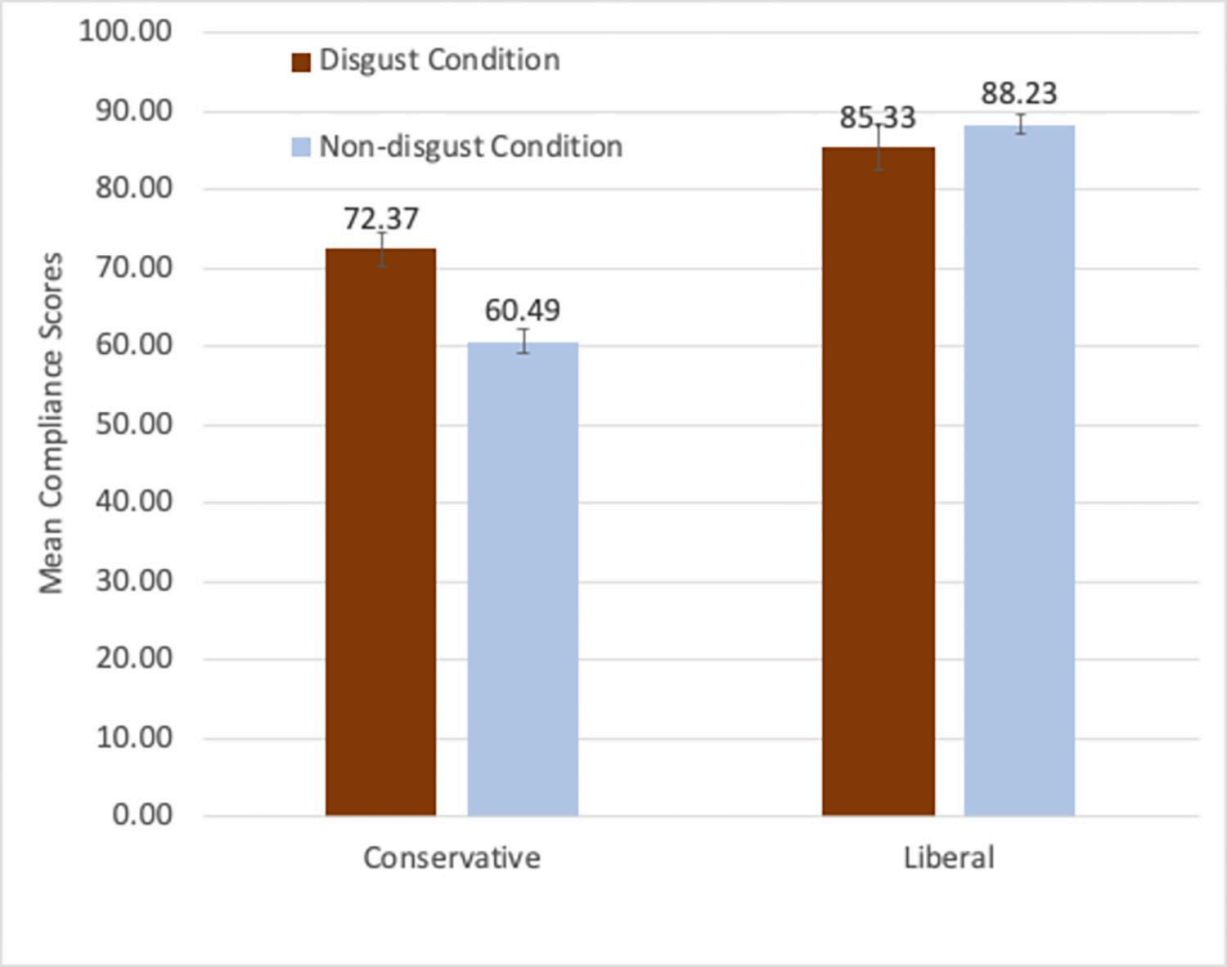
Mean compliance scores of the conservative and the liberal participants in the disgust and the non-disgust conditions of study 1. (Note: Error bars show standard errors).

Next, we tested whether the compliance scores were higher in the disgust condition than in the non-disgust condition, and whether this effect differed between conservative and liberal participants. The means of the four conditions are shown in Fig 1. A 2 (condition: disgust vs. non-disgust) X 2 (political orientation: liberal vs. conservative) ANOVA was carried out on these compliance scores. There was a significant main effect of political orientation: liberals’ compliance scores (*M* = 86.79, *SD* = 14.17) were significantly higher than conservatives’ (*M* = 66.42, *SD* = 23.81), *F*(1, 374) = 110.19, *p* < .001, partial *η*^2^ = .23. The compliance scores of the disgust condition (*M* = 79.71, *SD* = 18.93) were significantly higher than those of the non-disgust condition (*M* = 76.12, *SD* = 23.64), *F*(1, 374) = 5.19, *p* = .023, partial *η*^2^ = .014, but this main effect of condition was qualified by a significant interaction effect, *F*(1, 374) = 14.28, *p* < .001, partial *η*^2^ = .04.

The significant interaction effect was obtained because among conservatives, the compliance scores were significantly higher in the disgust condition (*M* = 72.37, *SD* = 20.21) than the non-disgust condition (*M* = 60.49, *SD* = 25.66), *t*(163) = 3.24, *p* = .001, whereas among liberals there was no significant difference between the disgust (*M* = 85.33, *SD* = 15.81) and the non-disgust condition (*M* = 88.23, *SD* = 12.23), *t*(211) = 1.49, *p* = .136. Sex of participant did not interact with any of the effects of the independent variables. (As detailed in the S1 File, regression analyses treating the liberalism-conservatism as a continuous independent variable and including those participants who were neutral on this dimension found the similar pattern of results.)

## Study 2

Study 2 was similar to Study 1 but was conducted after the COVID-19 vaccine started becoming available to the general public. As vaccines became more accessible, there was growing media attention to the perks offered with vaccination. Study 2 both replicated Study 1 and tested the effectiveness of these perks by adding a third condition where participants viewed images of perks currently used to encourage the general public to receive COVID-19 vaccination.

The dependent measures for compliance were the same as in Study 1, but to measure vaccine acceptance in the vaccine-perks condition, we separately examined participants’ willingness to receive COVID-19 vaccines only among those who had not yet been vaccinated. Because being vaccinated is a rather serious commitment, we predicted that it is unlikely that the perks, such as stickers or donuts, offered after vaccination would be enticing enough to those who are already reluctant to be vaccinated. Thus, we hypothesized that the disgust condition would result in the highest vaccination likelihood ratings of all three conditions.

As Study 2 introduced the vaccine-perks condition, the main focus of Study 2 was on those who had not yet been vaccinated. In fact, those who had received at least one dose of COVID-19 vaccine were more likely to be already compliant with the CDC’s public health guidelines. Therefore, the disgust manipulation may not be as effective among this population due to ceiling effects. The study was preregistered at https://osf.io/24fek and the data have been deposited at https://osf.io/t7d6a/.

### Methods

#### Participants

From March 29^th^, 2021 through April 8^th^, 2021, data from 1650 participants was collected. The target sample size was preregistered based on the following estimates. An adequate sample to detect the effect found among conservative participants in Study 1 with 80% power was 74 participants in each condition. Because Study 2 examines the effect of vaccine perks on people’s willingness to get vaccinated, we also calculated the target sample size based on Study 1’s results on conservative participants’ willingness to be vaccinated, finding an adequate sample size of 225. In addition, those who had been already vaccinated had to be excluded from the willingness to be vaccinated analyses. Considering about 10~20% vaccine rate at the time of data collection, we recruited 250 liberal participants in each of the 3 conditions. We also noticed from Study 1 that many pre-declared conservative participants reported that they were no longer conservative (more so than liberal participants did), and thus recruited 300 conservative participants in each of the 3 conditions.

Of the 1650 participants, 1469 were collected through Mechanical Turk in the same manner as in Study 1. Those who had participated in Study 1 were not allowed to participate in Study 2. Unfortunately, collecting a large number of pre-declared conservative participants through Mechanical Turk, as pre-registered, posed serious challenges. Because the study examines a time-sensitive issue, instead of prolonging the data collection process which would create variance caused by time lags, 181 participants (mixture of conservative and liberals) were collected through CloudResearch’s Prime Panel to reach the pre-registered targeted sample size of 1650. There was no significant interaction effect between these two groups of participants.

Of these 1650, 27 participants’ data were excluded for being incomplete, and 137 for failing the attention check explained in Study 1. Of the remaining participants, 254 participants’ political orientation, calculated in the same manner as in Study 1, was neither conservative nor liberal, and were excluded from the analyses. (See the S1 File for the analyses including these participants; In short, the pattern of the results did not change.)

Of the 1232 participants whose data were used for the analyses, 589 were classified as being conservative, and 643 as liberal; and 757 indicated that they had not received any COVID-19 vaccines, and 475 indicated that they received at least one COVID-19 vaccine shot. The participants were 84.1% White, 6.3% Asian, and 6.5% Black; 52.4% female; 37.9% had received a high school degree or some college education, and 44.2% received a bachelor’s degree. None of these demographic variables interacted with any of the independent variables, so these variables are not included in the analyses.

The study was approved by Yale University IRB. Participants provided written consent.

#### Materials, design, and procedure

Participants were randomly assigned to the disgust condition (n = 394), the non-disgust condition (n = 381), or the vaccine-perk condition (n = 457). The stimuli for the disgust and the non-disgust conditions were the same as in Study 1. The stimuli for the vaccine-perk condition were 5 perks that are currently being used to encourage Americans to receive vaccines. These included a discount at an upscale restaurant (https://www.oakandreel.com/), a free (legal) marijuana joint (https://www.greenhousemi.com/), a free Krispy Kreme donut (https://www.krispykreme.com/promos/vaccineoffer), a craft beer for 10 cents (https://www.marketgardenbrewery.com/), and an “I got my COVID-19 vaccine” button (https://www.wackybuttons.com/button-store/coronavirus/support-safety/i-got-my-covid-19-vaccine-white). As in the other two conditions, each stimulus consisted of one headline, which described the free or discounted item, and an image of the perk.

The procedure and the dependent measures were identical to those in Study 1 except for the following. Participants in the vaccine-perk condition were asked, after viewing all 5 trials, which of the vaccine perks they would choose to receive with their COVID-19 vaccine. All participants were also asked whether they received (1) first dose of Pfizer or Moderna, (2) both doses of Pfizer or Moderna or single dose Johnson and Johnson, or (3) none of the above. Those who chose (3) were the 757 participants who were classified as not having been vaccinated in the subsequent analyses.

### Results

Across all participants, the sub-questions within each of the indoor mask-wearing, outdoor mask-wearing, and the handwashing set all had high reliabilities (all Cronbach’s alpha’s > .90). Thus, for each participant, the average score within each of these sets was computed. These three average scores and the fourth and the fifth dependent measures (importance of frequent COVID-19 testing and likelihood of receiving COVID-19 vaccination) showed a high reliability (Cronbach’s alpha = .85) among those who had not been vaccinated. The five scores were averaged for each of these participants (see Table 2) and are henceforth called compliance scores, as in Study 1. Among those who had received at least one dose of vaccine, the first four dependent measures, excluding the likelihood of receiving vaccination, showed high reliability, Cronbach’s alpha = .78, and thus these four measures were averaged as their compliance scores.

**Table 2:** Descriptive statistics for the four sets of dependent measures for unvaccinated conservative and liberal participants in each condition in study 2.

Conservative						
Dependent Measures	Disgust		Non-disgust		Vaccine-perks	
	Condition		Condition		Condition	
	*M*	*SD*	*M*	*SD*	*M*	*SD*
Wearing masks indoors	74.44	31.08	69.83	32.86	66.95	33.75
Wearing masks outdoors	50.40	36.18	41.88	36.07	39.28	33.75
Cleaning hands	82.01	21.50	78.22	21.71	75.30	23.81
Testing for COVID-19	65.90	34.07	57.25	35.77	55.25	36.14
Liberal						
Dependent Measures	Disgust		Non-disgust		Vaccine-perks	
	Condition		Condition		Condition	
	*M*	*SD*	*M*	*SD*	*M*	*SD*
Wearing masks indoors	93.93	11.46	94.25	12.94	92.28	13.60
Wearing masks outdoors	79.96	22.86	80.54	22.18	74.88	25.55
Cleaning hands	87.67	15.35	87.00	15.79	86.92	15.28
Testing for COVID-19	87.78	20.50	90.55	17.44	87.35	20.10

**Fig 2:**
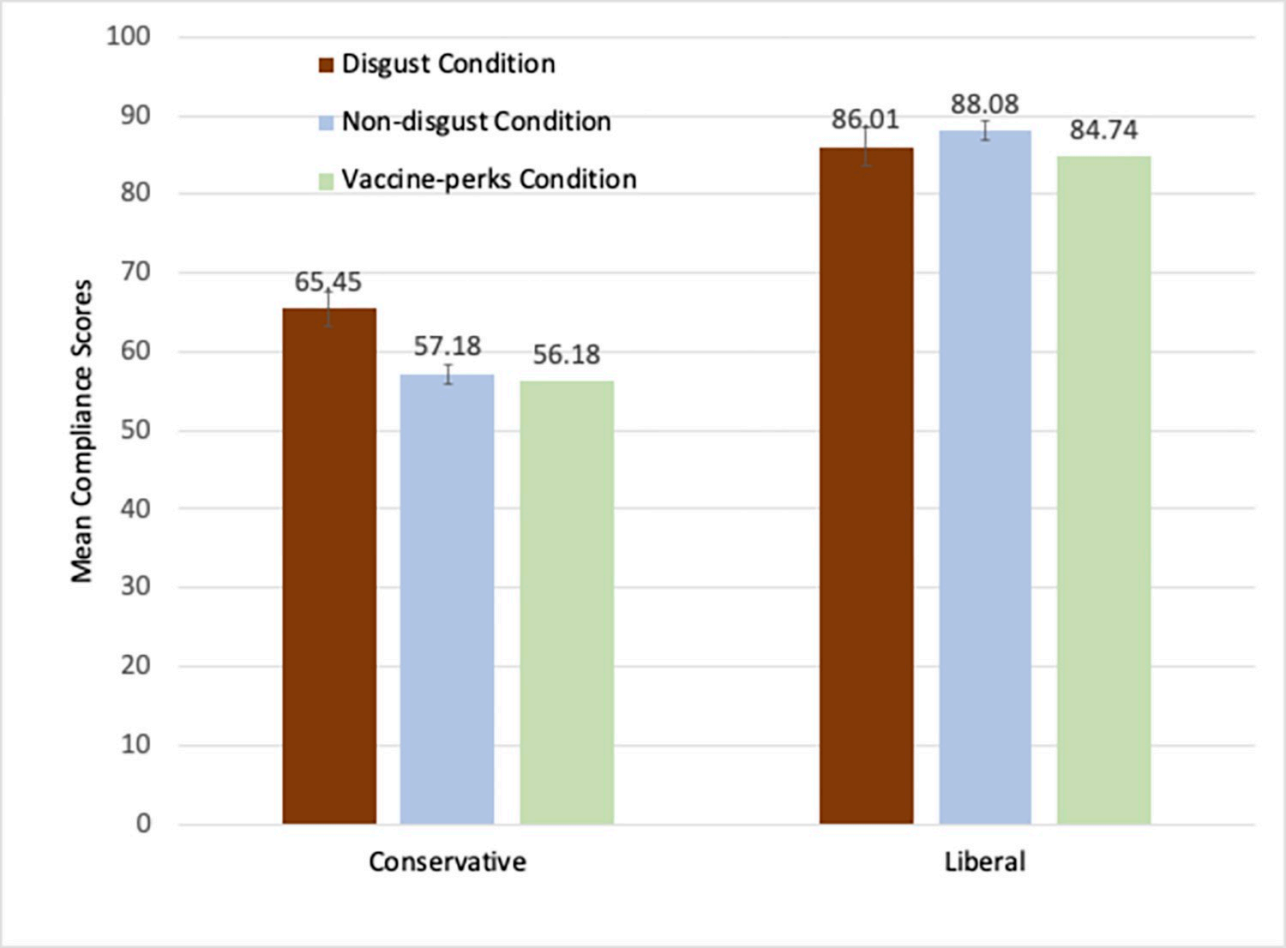
Mean compliance scores of the unvaccinated conservative and the liberal participants in the disgust, non-disgust, and the vaccine-perks conditions in study 2. (Note: Error bars show standard errors).

We first examined the results from those who had not been vaccinated. The mean compliance scores of the six conditions for these participants are shown in Fig 2. A 3 (condition: disgust, non-disgust, vaccine-perks) X 2 (political orientation: liberal vs. conservative) ANOVA was carried out on the compliance scores. There was a significant main effect of political orientation: liberals’ compliance scores (*M* = 86.18, *SD = 13*.*05*) were significantly higher than conservatives’ (*M* = 59.62, *SD =* 26.26), *F*(1, 751) = 306.29, *p* < .001, partial *η*^2^ = .29. There was a main effect of condition, *F*(2, 751) = 4.19, *p* = .016, partial *η*^2^ = .011, but this main effect of condition was qualified by a significant interaction effect, *F*(2, 751) = 4.15, *p* = .016, *η*^2^ = .011.

One-way ANOVAs with condition as a between-subject factor were carried out for each group of unvaccinated participants to understand the pattern of the significant interaction effect. Among the liberal participants, there was no significant effect of condition, *F*(2,355) = 1.99, *p* = .14. In contrast, among the conservatives, there was a significant main effect of condition, *F*(2,396) = 5.19, *p* = .006, *η*^2^ = .026. Because it was hypothesized that the compliance scores would be the highest in the disgust condition, planned independent samples t-tests are carried out comparing each of the two other conditions against the disgust condition. The compliance scores were significantly higher in the disgust condition (*M* = 65.45, *SD* = 25.27) than the non-disgust condition (*M* = 57.18, *SD* = 26.52), *t*(255) = 2.56, *p* = .011, replicating the results of Study 1. In addition, the scores in the disgust condition were significantly higher than the vaccine-perks condition (*M* = 56.18, *SD* = 26.20), *t*(275) = 3.00, *p* = .003. There was no difference between non-disgust and the vaccine-perks conditions, *p* > .99. Thus, there was no overall effect of priming for the liberal participants, and the disgusting images were the most effective way of increasing compliance among the conservative participants. Sex of participant did not interact with any of the effects of the independent variables. (See the S1 File for the regression analyses treating the liberalism-conservatism as a continuous independent variable and including those participants who were neutral on this dimension.)

**Fig 3:**
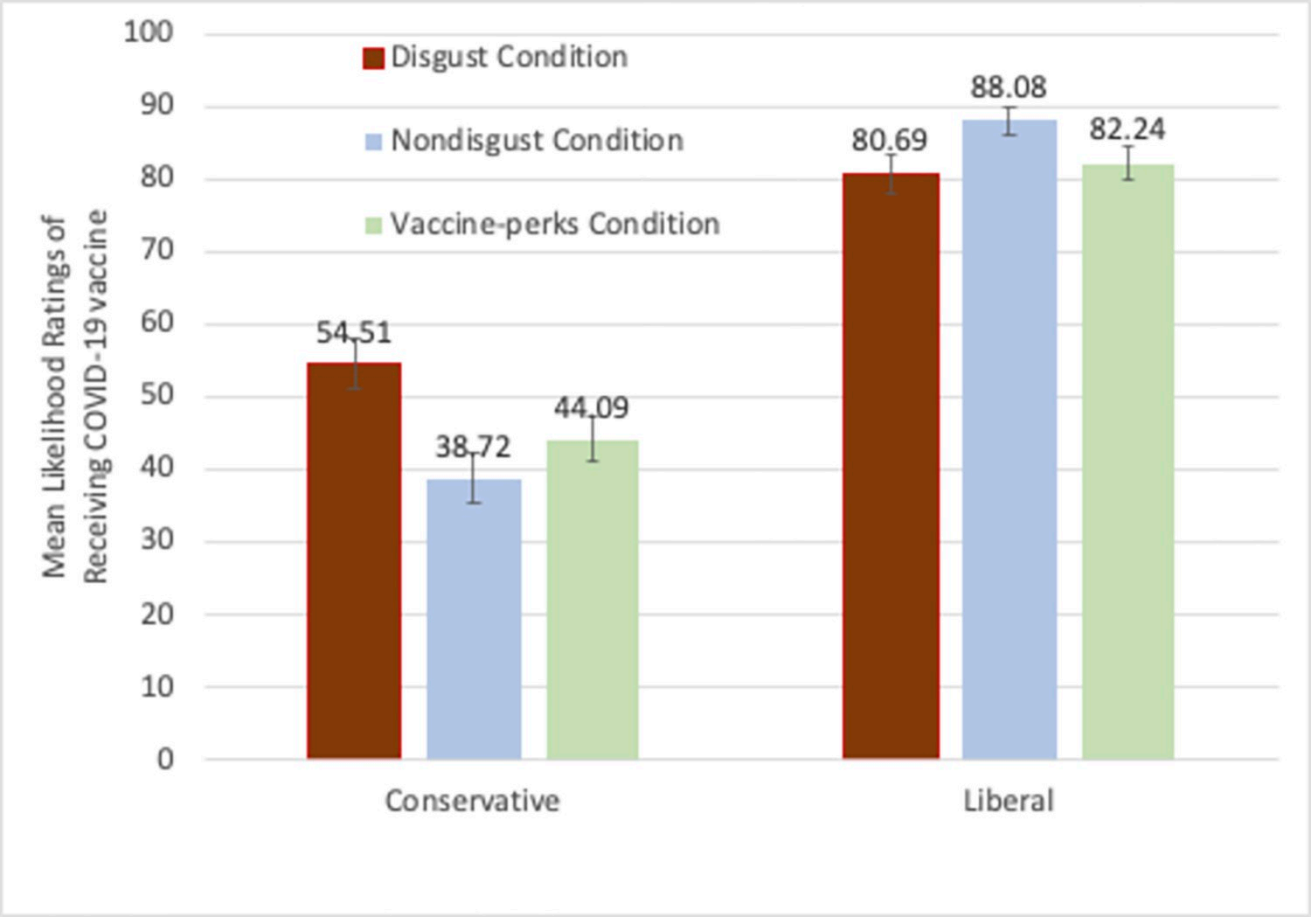
Mean Likelihood ratings of receiving COVID-19 vaccine by the unvaccinated conservative and the liberal participants in the disgust, non-disgust, and the vaccine-perks conditions in study 2. (Note: Error bars show standard errors).

Because the vaccine-perks are used to increase the likelihood of receiving vaccines, we next examined those ratings separately. For those who had not been vaccinated, the mean ratings of the six conditions are shown in Fig 3. A 3 (condition: disgust, non-disgust, vaccine-perks) X 2 (political orientation: liberal vs. conservative) ANOVA was carried out on the likelihood of receiving vaccination. There was a significant main effect of political orientation: liberals’ vaccination likelihood ratings (*M* = 85.53, *SD =* 26.27) were significantly higher than conservatives’ (*M* = 45.97, *SD =* 38.54), *F*(1, 751) = 247.89, *p* < .001, partial *η*^2^ = .25. There was no significant main effect of condition, *F*(2, 751) = 1.45, p = .236. There was a significant interaction effect, *F*(2, 751) = 7.44, *p* = .001, *η*^2^ = .019.

One-way ANOVAs with Condition as a between-subject factor were carried out for each group of unvaccinated participants to understand the pattern of the significant interaction effect. Among the liberal participants, there was no significant effect of condition, *F*(2,355) = 2.52, *p* = .08. In contrast, among the conservatives, there was a significant main effect of Condition, *F*(2,396) = 5.78, p = .003, *η*^2^ = .028. Planned independent samples t-tests showed that the vaccine likelihood scores were significantly higher in the disgust condition (*M* = 54.51, *SD* = 39.26) than the non-disgust condition (*M* = 38.72 *SD* = 38.24), t(255) = 3.26, *p* = .001. Most importantly, conservative participants in the vaccine-perk condition (*M* = 44.09, *SD* = 36.80) responded that they would be significantly less likely to receive COVID-19 vaccine than those in the disgust condition, *t*(275) = 2.28, *p* = .023. Thus, among the conservative participants, the disgusting images were the most effective way of increasing the likelihood of receiving COVID-19 vaccines.

The unvaccinated participants in the vaccine-perk condition were asked which perks they would prefer if they were to receive COVID-19 vaccine. Regardless of political orientation, Krispy Kream donuts were the top choice (31.1%), followed by 50% off a tasting menu at an upscale restaurant (21.6%), a free rolled marijuana joint (16.5%), and none of the above (15.8%).

Although the main focus of Study 2 was on those who have not been vaccinated, the data from those who had already been vaccinated were also examined. For these participants, compliance scores had to be calculated by averaging only the four sets of dependent measures excluding the likelihood of getting vaccinated. A 3 (condition: disgust, non-disgust, vaccine-perks) X 2 (political orientation: liberal vs. conservative) ANOVA was carried out on these compliance scores, and found only the main effect of political orientation, *F*(1,469) = 57.881, *p* < .001, partial *η*^2^ = .11 because the conservative participants were significantly less compliant (*M* = 73.99, *SD* = 20.76) than the liberal ones (*M* = 85.80, *SD* = 13.19). As alluded to earlier, those who had been vaccinated, even though conservative, were somewhat more compliant than those who had not been, diluting the effect of the disgusting images.

## Discussion

The present findings show that priming disgust improves intent to comply with public health guidelines. This effect was found only among conservatives, likely because conservatives view purity violations as moral transgressions more frequently than liberals do [17] and because pathogen disgust sensitivity predicts social conservatism [22]. Future research should investigate the mechanism of our findings. It is possible that, for example, disgusting images both prime disgust and prime fear about the severity of the disease and that these two factors together contribute to the mechanism behind our effect. However, the purpose of this study was to investigate a disgust intervention as a method for improving public health compliance; the question of why our intervention works is one for future work to explore.

The lack of effect of disgust versus non-disgust pictures among liberals could also be due to a ceiling effect, as liberals’ compliance scores were close to the maximum score of 100. Thus, we did not see a statistically significant effect among liberals because their compliance was already so high. However, the goal of this study was not to show that disgust does not affect liberals; rather, it was to test a potential public health intervention to overcome ideological barriers. Because liberals’ public health compliance intent was already high and did not decrease when disgust was primed, the intervention is not compromised among liberals and therefore still has potential to increase overall public health compliance.

Therefore, this research has direct applicability to the current COVID-19 pandemic: public health messaging could be made more effective, especially in U.S. counties with high concentrations of conservatives which are less compliant with social distancing [21], by including images that induce disgust. A disgust-inducing public announcement similar to the Tips campaign may have a significant positive impact on rates of social distancing, mask-wearing, and vaccine acceptance, all of which are crucial for the vaccines to effectively help us reach herd immunity even as the pandemic surges. The intervention we present could be used across news websites, social media platforms, and in online campaigns by public health agencies, especially in predominantly conservative counties. And as with the Tips campaign, the intervention does not appear to require a high-level of education. Additionally, we found that offering the vaccine perks that are currently popular did not increase the intent of liberals or conservatives to receive the COVID-19 vaccine. However, the perks did not increase vaccine hesitancy, ruling out the possibility [32] that incentives to ostensibly encourage people to get vaccinated is unlikely to make people view the vaccines as more dangerous and therefore be less likely to receive a shot. While potentially creating a challenging financial burden for individual businesses (such as a free donut every day for a year to hundreds of millions of Americans), the incentives appear to have no negative impact from a public health standpoint. In fact, they very well may convey benefits to those who receive them other than immunity to COVID-19, as was the case when New York City offered $100 to people who received their booster shot. The financial benefit of such a perk is significant for many individuals.

One limitation of the current study is that our sample is heavily skewed towards white participants. It remains to be seen whether disgusting images would be as effective in Asian, Black, and Latinx populations. Future studies should examine the nature of vaccine hesitancy and public health behavior in BIPOC communities specifically.

The current study also did not measure whether the disgust priming may increase a type of utilitarianism that permits instrumental harm for the greater good. In [15], it was found that pathogen disgust was associated with more willingness to kill an individual to save others, which can be translated as increased willingness to remove COVID-19 infected individuals from the society to save others. Our study did not measure the effect of disgust priming on this “instrumental harm” dimension [23]. Instead, we only measured taking positive actions for the greater good. Yet, judgments that permit instrumental harm are separate from judgments that impartially favor the greater good, and these two valences are actually independent of one another in the general population [23]. Thus, our finding that disgust priming increased positive utilitarianism (e.g., protecting others from being infected) does not necessarily imply that it would also increase negative utilitarianism (the willingness to harm some in order to protect others). Nonetheless, further studies should examine this possibility before utilizing the disgust images as an intervention against COVID-19.

Additionally, it is yet unclear whether this disgust priming method will directly translate to actual behavioral change. Our experiment investigated only the impact on self-reported behavior predictions, not behavior itself. Because it is difficult to examine everyday behavior in a controlled experiment setting, we recommend that future research study behavior in a field experiment—monitoring social distancing behavior (or perhaps even tracking infection with SARS-COV-2 itself) among individuals who are exposed to disgusting public health news images and comparing to that of individuals who are exposed to non-disgusting photographs. Nonetheless, given that moral emotions are powerful drivers of action [3], the present finding that a disgust intervention works on attitudes means that this moral emotion is likely to materialize as behavior change which is beneficial to society. Disgust interventions could have a positive impact on compliance with mitigation measures at a critical juncture in the U.S.’s COVID-19 pandemic response.

## Supporting information

S1 File Additional statistical analyses. (PDF)
