# Supplementary material for: It’s Time to be disgusting about COVID-19: Effect of disgust priming on COVID-19 public health compliance among liberals and conservatives
Source: PLoS One. 2022 May 12;17(5):e0267735. doi: 10.1371/journal.pone.0267735 (PMC9098091; doi:10.1371/journal.pone.0267735)
Supplement: S1 File — (PDF) [file pone.0267735.s001.pdf]

Supplementary Materials: Additional Statistical Analyses

In this section, we report additional statistical analyses performed. For Study 2, these analyses were not planned in the pre-registration of the study, but they were conducted at the request of the reviewers.

Regression Analyses Treating the Liberalism-conservatism as a Continuous Variable

Instead of excluding the participants who were neither liberal nor conservative, regression analyses were carried out including all participants who passed the attention check while treating the liberalism-conservatism as a continuous variable. The results found from these analyses are consistent with the main conclusions drawn in the main text.

Specifically, participants' ratings on their political party (i.e., strong Democrat to strong Republican) and on their political ideology (i.e., strong liberal to strong conservative) were added to yield the composite, continuous score of their liberalism-conservatism. For Study 1, a multiple linear regression was calculated to predict the total compliance score based on the composite liberalism-conservatism score, the condition, and the interaction between the composite score and the condition. A significant regression equation was found, $F(3, 389) = 47.73$, $p<.001$, with an R^2 of .27. The condition (standardized $\beta1 = -237$, $p=.009$), the liberalism-conservatism (standardized $\beta2 = -.654$, $p<.001$), and the interaction term (standardized $\beta3 = .398$, $p<.001$) significantly predicted the compliance score.

For Study 2, a similar multiple linear regression was calculated to predict the total compliance score based on the composite liberalism-conservatism score, the condition, and the interaction between the composite score and the condition. A significant regression equation was found, $F(3, 884) = 105.64$, $p<.001$, with an R^2 of .26. It was found that the liberalism-conservatism (standardized $\beta2 = -.552$, $p<.001$) significantly predicted the compliance score. However, the condition and the interaction term were not significant predictors of the compliance score, p's > .110. However, if the condition was restricted to the Non-Disgust and the Disgust conditions (i.e., excluding the vaccine-perks condition) as in Study 1, the results replicated those in Study 1. Specifically, the same multiple linear regression was computed with only the participants in the Disgust and the Non-Disgust conditions. A significant regression equation was found, $F(3, 564) = 71.89$, $p<.001$, with an R^2 of .28. The condition (standardized $\beta1 = -176$, $p=.034$), the liberalism-conservatism (standardized $\beta2 = -.625$, $p<.001$), and the interaction term (standardized $\beta3 = .269$, $p=.003$) significantly predicted the compliance score.

Incomplete Data

In Study 2, 27 out of 1650 who reached the end of the survey skipped more than 15% of the responses (1.3%, 1.6%, and 1.9% of the Disgust, Non-Disgust, and vaccine condition, respectively). The Fisher's exact test showed no significance differences between each pair of the conditions, all p's > .10. These data were treated as being "incomplete" and not included in the analyses.

Participants Who Could Not Be Classified as Either Liberal or Conservative

In study 1, 16 participants could not be classified as either liberal or conservative and were excluded from the analyses reported in the main text. There was no significant difference between the two experimental conditions in terms of the proportion of these participants (4.6% in the Disgust condition, and 3.4% in the Non-Disgust condition, Fisher's exact test $p > .10$).

In Study 2, 20.0% of the participants could not be classified as either liberal or conservative and were excluded from the analyses reported in the main text. There was no significant difference between the Disgust condition (16.4%) and the Non-Disgust condition (19.2%) in terms of the proportion of these participants, $X^2(1, N = 998) = 1.37$, $p = .24$. The vaccine-perk condition (23.2%) did not differ from the Non-Disgust condition, $X^2(1, N = 1124) = 2.59$, $p = .11$. but significantly differed from the Disgust condition, $X^2(1, N = 1124) = 7.97$, $p = .005$.

Participants Who Failed the Attention Check

At the end of the survey in both studies, participants were re-presented with the five images they saw earlier and the five foils (i.e., images that participants in the other experimental condition saw) in a randomized order and were asked to determine whether they had seen each image in the earlier part of the study. Participants' recognition memory was scored by adding 1 point for each "old" image they judged as having been presented and subtracting 1 point for each "new" image that they judged as having been presented. Thus, the score could range from -5 to +5. Those who scored 2 or lower were classified as having failed the attention check and were excluded from the analyses reported in the main text.

In Study 1, the number of participants who failed the attention check did not differ between the conservative (1.2%) and the liberal participants (1.4%). However, there were more participants who failed the attention check in the Non-Disgust condition (2.6%) than in the Disgust condition (0.0%) according to Fisher's exact test, $p < .05$. To ensure that the results reported in the main text were not biased due to this difference, we also carried out the analyses reported in the main text including those who failed the attention check, and found that the conclusions drawn from the analyses reported in the main text remain the same. More specifically, a 2 (condition: Disgust vs. Non-Disgust) X 2 (political orientation: liberal vs. conservative) ANOVA was carried out on the compliance scores of the participants regardless of whether they passed or failed the attention check. There was a significant main effect of political orientation: liberals' compliance scores ($M = 86.59$, $SD = 14.23$) were significantly higher than conservatives' ($M = 66.58$, $SD = 23.71$), $F(1, 379) = 106.73$, $p < .001$, partial $\eta^2 = .22$. The compliance scores of the Disgust condition ($M = 79.73$, $SD = 18.93$) were significantly higher than those of the Non-Disgust condition ($M = 76.10$, $SD = 23.39$), $F(1, 379) = 5.22$, $p = .023$, partial $\eta^2 = .014$, but this main effect of condition was qualified by a significant interaction effect, $F(1, 379) = 12.77$, $p < .001$, $\eta^2 = .033$.

In Study 2, among those who had not been vaccinated, which was the main focus of the study, the conservative participants (4.5%) were more likely to fail in the attention check than the liberal participants (1.9%), $p=.046$ according to Fisher's exact test. The three experimental conditions did not significantly different from each other (3.1% in the Disgust condition, 5.3% in the Non-Disgust condition, and 1.8% in the Vaccine-perk condition), $p>.08$. To ensure that the

results reported in the main text were not due to difference in the exclusion rate between the conservative and liberal participants, we carried out the analyses reported in the main text including those who failed the attention check and found that the conclusions drawn from the analyses reported in the main text remain the same. More specifically, a 2 (condition: Disgust, Non-Disgust, Vaccine-Perk) X 2 (political orientation: liberal vs. conservative) ANOVA was carried out on the compliance scores with these participants. There was a significant main effect of political orientation: liberals' compliance scores (M = 85.90, SD = 13.29) were significantly higher than conservatives' (M = 59.57, SD = 26.24), $F(1, 777) = 305.55$, $p < .001$, partial $\eta^2 = .28$. There was a significant main effect of condition, $F(2, 777) = 3.42$, $p = .033$, partial $\eta^2 = .009$, but this main effect of condition was qualified by a significant interaction effect, $F(2, 777) = 3.90$, $p = .021$, $\eta^2 = .010$.
